# iJobs – An online implementation of the JOBS II program for fostering reemployment: A feasibility and acceptability study

**DOI:** 10.1016/j.invent.2023.100674

**Published:** 2023-09-21

**Authors:** Alexandra Bodnaru, Andrei Rusu, Roland W.B. Blonk, Delia Vîrgă, Dragoș Iliescu, Anja Van den Broeck

**Affiliations:** a Department of Psychology, West University of Timișoara, Romania; b Department of Human Resource Studies, Tilburg University, the Netherlands; c Department of Psychology and Cognitive Sciences, University of Bucharest, Romania; d Department of Work and Organization Studies, KU Leuven, Brussels, Belgium; e Optentia, North-West University, Vanderbijlpark, South Africa

**Keywords:** iJobs, JOBS II, Internet intervention, Unemployment, Feasibility trial

## Abstract

The current study aimed to test the feasibility and acceptability of iJobs, an online adaptation of the JOBS II program (Curran et al., 1999). iJobs is a two-week internet intervention for the unemployed, consisting of five modules. This study is an open-label trial with an uncontrolled, within-group, pre-posttest, and follow-up design. Out of the 56 participants allocated to the intervention, 36 completed (M_age_ = 25 years; 57.1 % females) the post-test (36 % dropout), and 34 the three months follow-up. The protocol-compliant participants followed the modules with great engagement (mean quality of assignments completion above 4 points out of 5 for each module). The online platform's usability was high (84.86 points out of 100). Participants reported high overall satisfaction with the program. Our results suggest that iJobs is a feasible intervention and was accepted by its beneficiaries. Relative to baseline, inoculation against setbacks (*d* = 0.64), job search self-efficacy (*d* = 0.50), and self-esteem (*d* = 0.28) increased significantly, while future career anxiety in the COVID-19 context decreased significantly (*d* = 0.34). No significant differences were found for depression, anxiety, and job-search behaviors. At three months follow-up, 55.9 % of the participants found employment, 5.9 % were in a job selection process, and 38.2 % were still unemployed. Job satisfaction was high among the employed.

## Highlights

• Access to specialized interventions is hindered for many unemployed in need of assistance.
• Scalable evidence-based interventions for fostering employment are highly needed.
• iJobs is a feasible and acceptable online adaptation of the JOBS II job search intervention.
• Evidence for causal inferences regarding efficacy and effectiveness is needed.

## 1 Introduction

Unemployment is a prevalent phenomenon, affecting hundreds of millions worldwide (Statista, 2023a). In the past couple of years, the unemployment rate increased because of the COVID-19 crisis while its asymmetrical spread accentuated (e.g., in the EU, the unemployment rate was between 2.7 % in Czechia and 12.7 % in Spain, as of April 2023; Statista, 2023b). Besides its economic repercussions, unemployment is also associated with decreased well-being (Wanberg et al., 2020), leading to mental health impairment (i.e., depression and anxiety) (Paul and Moser, 2009). Although the unemployed would benefit from work and health promotion programs, they are a hard-to-reach population. Therefore, scalable and easy-to-deliver evidence-based intervention programs for fostering employment are highly needed.

Job search interventions combine classical job application training with socio-psychological elements. One example is the JOBS II program (Curran et al., 1999), which intends to promote reemployment and prevent mental health among the unemployed. This is an evidence-based job search intervention that comprises five elements: *Job-search skills*, *Active learning* (through group discussions, brainstorming sessions, and role-playing exercises), *Inoculation against setbacks* (problem-solving skills and enhancement of coping strategies), *Social support* (from both the facilitators and the beneficiaries), and *Trainer referent power* (trainers build trust and offer specific positive feedback) (Curran et al., 1999). Hence, its structure makes it one of (if not the only) programs blending almost all of the critical components for effective job search interventions highlighted by Liu et al. (2014) in their meta-analysis. The JOBS program was developed at the Michigan Research Center in 1984, and its effectiveness was tested in large-scale trials across the US until the late '90s (Price and Vinokur, 2014). Over the years, the program has been adopted in different countries around the world: Finland (Vuori and Vinokur, 2005), China (Price et al., 2006), Ireland (Barry et al., 2006), Netherlands, Sweden, and South Korea (Price and Vinokur, 2014). Recently was used in South Africa (Paver et al., 2020), and a randomized controlled trial is ongoing in Germany (Hollederer et al., 2021). However, thus far, the program has been delivered solely in a face-to-face setting. The recent context of the COVID-19 pandemic showed us that sometimes directly delivering interventions may be impossible. Hence, a solution to this problem can be delivering such interventions over the Internet. The advantages of web-based interventions for the unemployed are that get around stigma and the cost barriers because they allow self-administration (if delivered asynchronously) and also have the potential to reach more beneficiaries quickly (high scalability). Internet interventions have already proved their efficacy, cost-effectiveness, and scalability in mental health (Ebert et al., 2018). Hence, our study aimed to adapt the JOBS II program to an online setting and test its feasibility and acceptability.

The JOBS program features various job search strategies and exercises, including role-playing informal interviews and acquiring networking techniques, to encourage both job-seeking intensity and quality. According to a recent literature review (Van Hooft et al., 2021), employment success and employment quality are predicted by the intensity and quality of job search behavior. *Job search intensity* refers to the effort and time dedicated to such activities (e.g., talking to others about job opportunities), while *job search quality* implies that the behaviors are performed systematically and well-prepared (e.g., networking, interview behavior, resumes) (Van Hooft et al., 2013, Van Hooft et al., 2021).

Unemployment is often associated with feelings of inferiority and incompetence (Curran et al., 1999). In this context, the program promotes change mechanisms such as coping resources (i.e., inoculation against setbacks), self-efficacy (i.e., job search self-efficacy), and self-esteem. Those mechanisms seem critical for the job search process to be successful (Paver et al., 2020; Price and Vinokur, 2014; Vinokur et al., 1995a, Vinokur et al., 1995b, Vinokur et al., 2000). *Inoculation against setbacks* refers to the individual's ability to cope successfully with stressful situations and develop strategies to overcome them (Vuori and Vinokur, 2005). During JOBS, participants identify the perceived barriers in the re-employment process and the appropriate strategies to overcome them. *Job search self-efficacy* refers to the individual's belief that they can perform job search activities successfully (Saks and Ashforth, 1999; Vinokur and Caplan, 1987). *Self-esteem* refers to the favorable or unfavorable perception of oneself (Rosenberg, 1979) and plays an important role in maintaining the job seeker's motivation and perseverance in the job search process (Caplan et al., 1989; Paver et al., 2020). JOBS enhances self-esteem by encouraging participants to acknowledge, expose and develop their personal resources (Paver et al., 2020), and intends to maximize participants' job search abilities (Price and Vinokur, 2014).

The unemployed experience more distress than the employed, due to financial strain and lack of other basic psychological needs, and might struggle with mental health issues, especially if the duration of unemployment is longer (Paul and Moser, 2009). Through its mechanisms, JOBS has not only short-term (Vinokur et al., 1995a, Vinokur et al., 1995b) but also long-term (Vinokur et al., 2000) positive effects on mental health and reduces depressive symptoms (Price et al., 2002; Vinokur et al., 1995a, Vinokur et al., 1995b; Vinokur et al., 2000). The unemployed also encounter fear associated with re-employment, which, is defined as career anxiety. In the recent context of the COVID-19 crisis, thousands of people lost their jobs, which directly impacted career anxiety (Lee et al., 2020; Mahmud et al., 2020).Therefore, career anxiety in the COVID-19 context might be an obstacle to re-employment that can be addressed within the JOBS program.

In the present study, we created an online version of the JOBS II program (referred to as iJobs) and conducted an open-label trial to test its feasibility and acceptability using a non-experimental design with repeated measures. iJobs is a two-week intervention comprising five sessions, each of them requiring 1 to 2 h for completion. First, we evaluated treatment adherence, satisfaction with the program, and the system's usability. Second, we expected that after the 2-week intervention, participants' job search self-efficacy (H1), inoculation against setbacks (H2), and self-esteem (H3) would increase, while depression (H4a), anxiety (H4b), and career anxiety in the COVID-19 context (H4c) symptoms would decrease. At the 3-month follow-up, we measured participants' job search behaviors, employment status, and job satisfaction (of those who secured employment).

## 2 Methods

### 2.1 Study design

The current study is a feasibility open trial with an uncontrolled, within-group, pre-posttest, and follow-up design.

### 2.2 Participants

Eligible participants were unemployed Romanian adults recruited via social media posts (i.e., posts on LinkedIn and about twenty Facebook groups for jobseekers from different country regions) and student mailing lists from Romanian universities via Career Counseling Centers (i.e., based in Timișoara, Bucharest, and Iași).

Inclusion criteria for the study were: (1) age between 18 and 60 years; (2) currently unemployed and looking for a job; (3) or work as a volunteer and looking for a paid job; and (4) access to a PC or laptop and essential digital competencies.

Exclusion criteria were: (1) not having Internet access and no availability during the two weeks of the program; (2) suffering from a chronic disease that implies special conditions for taking part in the activities; (3) moderately severe and above clinical depression symptoms (Cut-off = 15 at PHQ9; Kroenke et al., 2001); or (4) severe clinical anxiety symptoms (Cut-off = 15 at GAD7; Spitzer et al., 2006). Since this is a feasibility trial and the first attempt to adapt the intervention to an online version and the JOBS program is intended for non-clinical populations, participants with severe clinical depression or/and anxiety symptoms were included only if they also received mental health treatment (self-reported).

### 2.3 Procedure

An overview of the program with a link to open the iJobs platform was posted on social media and sent to student mailing lists. Those who wanted to participate had to create an account on the website. They gave informed consent, accepted the General Data Protection Regulation (GDPR) statement, and entered an email address. Afterward, they automatically received two emails: one for the email confirmation and the other with the login information. The username was a random combination of characters (e.g., 4418mwhl) to ensure anonymity. When participants accessed their accounts, they could complete the pre-test measures. Participants with high depressive or/and anxiety symptoms who did not receive psychological assistance were excluded and directed to a specialist in mental health. Eligible participants received an email with the schedule and brief instructions.

**Fig. 1:**
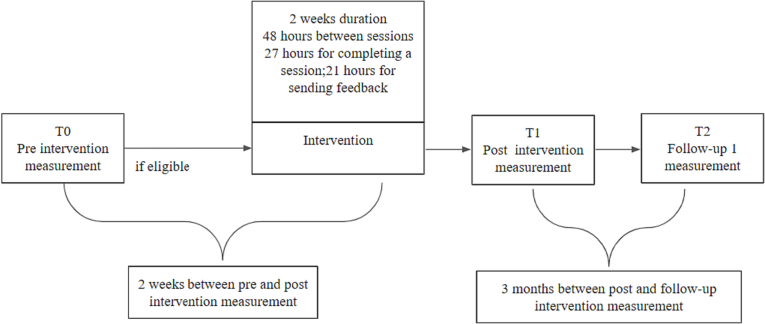
Procedure overview.

Each module was available for completion for 27 h. After that, the module became inaccessible. Before the next module, participants received personalized written feedback from the team of counselors (5 people) involved in the program. They also received an email when a new module was available. After the fifth module, the post-test questionnaires were administered on the platform. Three months after the intervention, participants who completed the post-test measures were contacted for a brief phone screening regarding their employment status, and job search behaviors. They could win a backpack or an insulated water bottle (raffle of 19 prizes) for participating in the follow-up data collection. The entire procedure is depicted in Fig. 1.

### 2.4 Intervention

iJobs is an online adaptation of JOBS II: *A manual for teaching people successful job search strategies* (Curran et al., 1999). It was adapted as a two-week intervention with 5 modules that focus on discovering job skills (Session 1), dealing with obstacles to employment (Session 2), finding job openings (Session 3), resumes, contacts, and interviewing (Session 4), the complete interview and planning for setbacks (Session 5). The content is text-type, and participants gave written answers to all exercises. An overview of the modules is in Table 1.

**Table 1:** Overview and brief descriptions of the iJobs modules.

Module name	Brief description
Discovering your job skills	Participants read an overview of the iJobs content in the opening module and established personal objectives. The module focuses on identifying their strengths and exercising ways of demonstrating them during interviews, using social learning theory (i.e., case studies for demonstrating effective and ineffective interviews).
Dealing with obstacles to employment	In the second module participants identify the obstacles and challenges they face in the employment process and exercise ways to address those during interviews, to defuse the employer's fears.
Finding job openings	The third module focuses on finding job openings through networking. Participants identify their extended network, learn about and practice informational interviews with people from their network. For such exercises, participants received a scenario in the form of a table (one column with questions or replicas) and the other column dedicated to their written answers.
Resumes, contacts, and interviewing	In the fourth module, participants analyze resumes and learn how to structure the information effectively. In the second part, participants use role-playing to practice the interview from the perspective of the employer and the candidate by answering predefined questions or replicas.
The complete interview and planning for setbacks	The final module helps participants integrate the information gained by exercising an entire interview. It also helps them anticipate and establish strategies for overcoming potential obstacles in the employment process.

iJobs is asynchronous, thus in contrast to JOBS II, participants did not communicate in real-time with a counselor or with each other during the intervention. Participants only interacted through messages with one of the counselors involved in the feedback process (the same person throughout the program). The sections dedicated to facilitators in the JOBS II manual were used to instruct the counselors about the iJobs' purpose, content, and role in the program: to offer social support and to increase participants' self-esteem and self-efficacy by giving constructive, positive feedback.

**Fig. 2:**
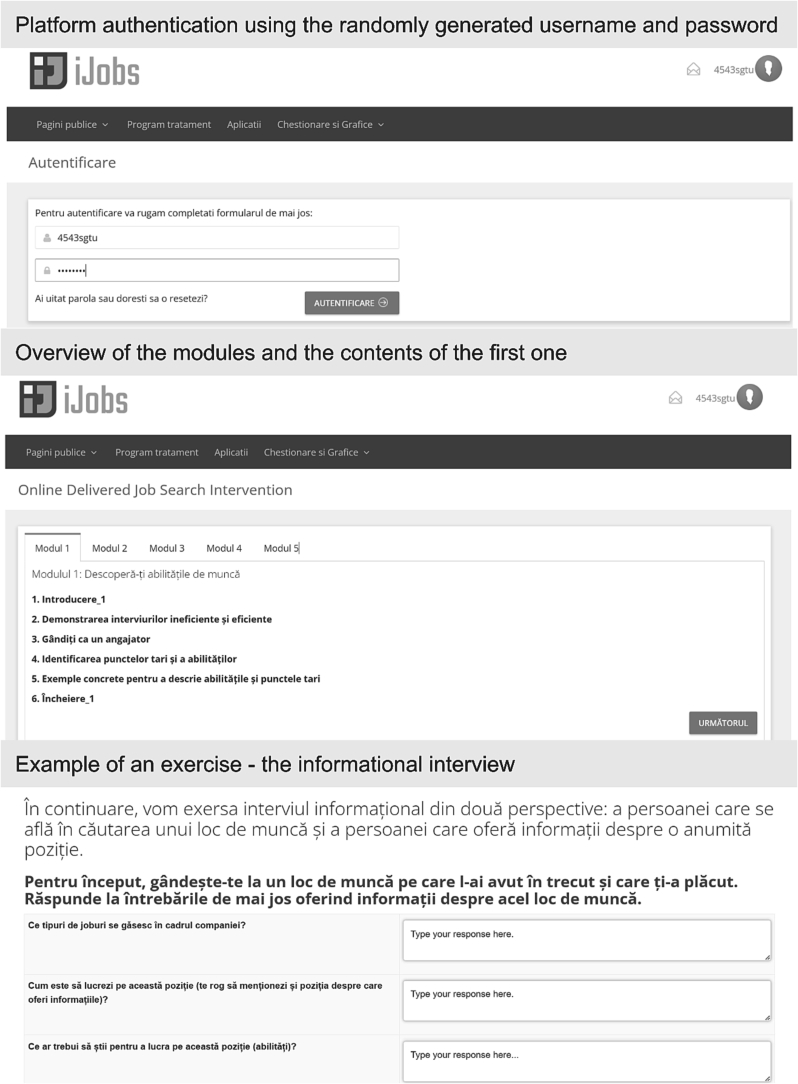
iJobs interface on the www.e-cbt.ro platform.

We used the www.e-cbt.ro platform to deliver iJobs, which was designed for and successfully used in other online mental health interventions (e.g., Isbășoiu et al., 2021; Tulbure et al., 2018). All the sensitive content is encrypted and stored on a secure server. The visual interface of the platform is displayed in Fig. 2.

### 2.5 Assessment measures

#### 2.5.1 Feasibility

*Adherence*. Treatment adherence was evaluated through the number and quality of completed assignments, using qualitative ratings ranging from 1 to 5 of the degree of completeness and depth of the answer for each assignment. Two independent experts conducted the assessment based on a priori established coding grids (i.e., “Number of complete, partially complete and incomplete assignments per session.”, “The participant understood the assignments.” <1 - not at all, 5 – yes completely>, “The participant completed the assignment by giving long, complex answers. <1 - not at all, 5 – yes completely>”).

#### 2.5.2 Acceptability

*Satisfaction with the intervention*. Satisfaction was measured at the end of the intervention using 22 items. Six items on a 7-point Likert scale aiming at the program's content (e.g., “This training will be useful to me in the future.”), retrieved from a questionnaire used for measuring the alliance between trainers and trainees in a face-to-face JOBS II intervention (Rusu, 2013). We used 9 items (e.g., “How would you evaluate the quality of the received information?”) on a 4, 5, and 10-point scale from Richards et al. (2016), previously used in questionnaires evaluating the satisfaction with interventions delivered on www.e-cbt.ro. Finally, there were also 7 open-ended questions (e.g., “The most valuable experience of this program was:”).

*Usability*. The System Usability Scale (SUS; Bangor et al., 2009) was used to measure participants' satisfaction with www.e-cbt.ro. It consists of 10 items (e.g., “I think this platform is easy to use.”) on a 5-point scale (α = 0.83). The scoring formula proposed by Brooke (1996) generates a total score between 0 and 100 (i.e., the sum of all the items is then multiplied by 2.5); a higher score indicates increased usability.

#### 2.5.3 Outcome measures

*Job search behavior*. We used a version of Blau's (1994) Job Search Behavior Scale adapted to Romanian job seekers and the contemporary context (JSB; Vîrga and Rusu, 2018) to assess participants' involvement in job search activities at baseline and follow-up. It consists of 16 items on a 5-point scale (α = 0.93), further divided into job search intensity (α = 0.91), with 13 items (e.g., “How often did you send your resume to potential employers?” or “How often did you use online social media networks to search for job openings?”), and job search effort (α = 0.94) with 3 items (“You have made every effort to find a job.”). The total score ranges between 13 and 65 for intensity and between 3 and 15 for effort (overall, it ranges between 16 and 80). A higher score indicates increased job search behavior.

*Job search self-efficacy*. Job Search Self-Efficacy Scale (JSSE; Saks et al., 2015) was used to assess participants' perception of their ability to gain employment at baseline and post-intervention. The scale consists of 20 items (e.g., “How confident you are in your ability to succeed in finding a job?”) on a 5-point scale (α = 0.95). The total score ranges between 20 and 100. A higher score means increased job search self-efficacy.

*Self-esteem*. Rosenberg's Self-esteem Scale (RSE; Rosenberg, 1979) assesses global self-worth at baseline and post-intervention. The scale consists of 10 items (e.g., “I feel that I have a lot of qualities.”) on a 4-point scale (α = 0.87). The total score ranges between 10 and 40. A higher score indicates increased self-esteem.

*Inoculation against setbacks*. Inoculation against setbacks was assessed at baseline and post-intervention. We used 2 out of 3 items (e.g., “Do you have a plan for possible obstacles during the job-seeking process?”) on a 5-point scale, retrieved from Vuori and Vinokur (2005), to measure participants' ability to deal with setbacks (α = 0.44). One item was excluded for internal consistency reasons. The total score ranges between 2 and 10. A higher score means a better outcome.

*Future career anxiety*. The Future Career Anxiety Scale (Mahmud et al., 2020) was used to assess participants' anxiety regarding their future careers in the context of the COVID-19 outbreak, both at baseline and post-intervention. The scale consists of 5 items (e.g., “I worry about future employment because of fierce competition in the job market due to the outbreak of COVID-19.”) on a 5-point scale (α = 0.84). The total score ranges between 5 and 25. A higher score indicates a worse outcome.

*Anxiety symptoms*. Generalized Anxiety Disorder Assessment (GAD-7; Spitzer et al., 2006) was used to assess the severity of anxiety symptoms in the past two weeks, according to the Diagnostic and Statistical Manual of Mental Disorders, Fifth Edition (DSM-V) criteria. The scale consists of 7 items (e.g., “In the past two weeks, how often have you had trouble relaxing?”) on a 5-point scale (α = 0.86). The total score ranges between 0 and 21. A higher score indicates a worse outcome.

*Depressive symptoms*. Patient Health Questionnaire-9 (PHQ-9; Kroenke et al., 2001) was used to measure participants' severity of depression symptoms in the past two weeks, according to DSM-IV criteria. The scale consists of 9 items (e.g., “In the past two weeks, how often you have felt tired or a lack of energy?”) on a 5-point scale (α = 0.79). The total score ranges between 0 and 27. A higher score indicates a worse outcome.

*Employment status*. Self-reported employment status was phone screened with a dichotomous question (i.e., “Do you have a paid job at the moment?”) at the 3-month follow-up.

*Job quality*. Job quality was phone screened for those who found employment with 3 questions (i.e., “Is your current job in the aimed field?” Y/N, “How much do you like your job on a 10 points scale?”, “How satisfied are you with your current salary on a 10 points scale?”) that assess the overall satisfaction with the secured job and salary.

#### 2.5.4 Additional measures^1^

*Socio-demographic information*. In order to evaluate the sample characteristics, we collected the following information: age, gender, residential area, educational level, average monthly income, unemployment period, work experience, and targeted professional field.

### 2.6 Sample size

The sample size estimation, performed in GPower (Faul et al., 2007) for paired samples *t*-tests aiming for a statistical power of 1-β = 0.80, a moderate effect size (*d* = 0.50), and a large intra-measures correlation (*r* = 0.60), was of 27 participants. Based on Melville et al. (2010) meta-analysis, we anticipated a dropout rate of 30 %. Thus, the final sample aimed for enrolment was 35 eligible participants.

### 2.7 Statistical analysis

Descriptive statistics were used to evaluate baseline demographics, feasibility, and acceptability outcomes. Paired sample *t*-tests were used to analyze the within-group differences. Per-protocol and intent-to-treat (ITT) analyses (complete-case analyses based on multiple imputation methods) were performed. The intent-to-treat analysis includes every participant allocated to the intervention, ignoring withdrawal and anything that happens with the participants after allocation, so overestimations of the efficacy of interventions are avoided (Gupta, 2011). This approach is a standard for randomized trials, and less for other types of designs (Gupta, 2011). In our context (of a high percentage of missing data), we performed ITT just as a secondary analysis to increase the robustness of the results and reduce potential biases of the estimates. Multiple imputations (Salim et al., 2008) were performed in R, using the MICE algorithm described by Van Buuren and Groothuis-Oudshoorn (2011) – each incomplete variable has a separate imputation model. Cohen's *d* with a 95 % confidence interval was used to calculate the within-group effect sizes. In addition, conventional qualitative content analysis (Hsieh and Shannon, 2005) was performed for the open-ended items.

### 2.8 Ethics and approval

All participants signed an informed consent form. This trial was approved by the Scientific Council of University Research and Creation from West University of Timisoara (No. 19260/22.04.2021) and registered in the ClinicalTrials.gov PRS (NCT04885400).

## 3 Results

### 3.1 Participants

**Fig. 3:**
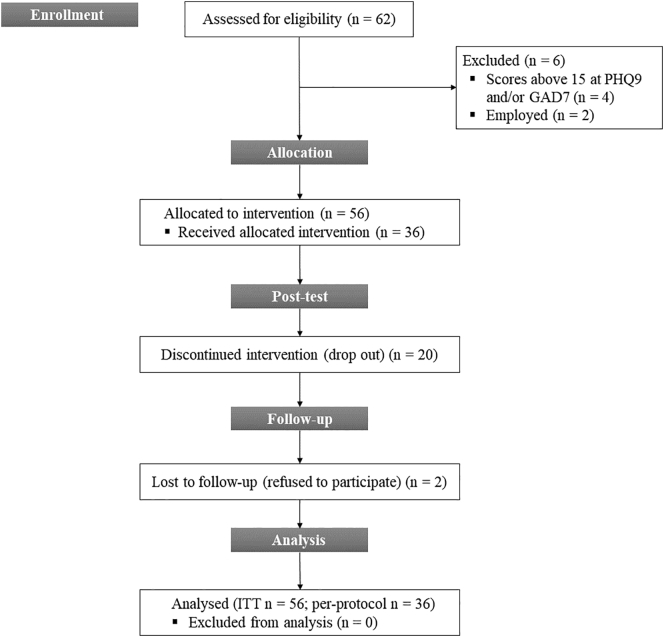
Adapted CONSORT diagram depicting participants' flow throughout the study.

During the two-and-a-half weeks of the recruitment period, 62 participants enrolled in the program. Out of them, 56 were allocated to the intervention, 36 completed the post-test, and 34 completed the three months follow-up (see Fig. 3 for the flow chart). As shown in Table 2, 57.1 % of participants were female, and their mean age was 25 years. There are no significant differences between completers and dropouts in terms of baseline characteristics (see Table 2).

**Table 2:** Socio-demographic sample characteristics.

Demographics	Total (*N* = 56)	Comparisons between completers (*n* = 36) and dropouts (*n* = 20)
Age *M(SD*)	25.69 (7.62)	*t*_(54)_ = 1.29, *p* = .204
Gender (%)	57.1 % female	χ^2^_(1)_ = 2.10, *p* = .147
Education (%)	Bachelor student/degree 42.8 % Master's student/degree 53.6 % Ph.D./Ph.D. student 3.6 %	χ^2^_(5)_ = 2.16, *p* = .827
Average monthly income (%)	Below 200 euros 7.1 % 200–400 euros 17.9 % 400–700 euros 23.2 % 700–900 euros 30.4 % Above 900 euros 21.4 %	χ^2^_(4)_ = 0.35, *p* = .986
Period of unemployment (Mdn; months)	5.50	*t*_(54)_ = 1.73, *p* = .091
Prior work experience (Mdn; months)	7.50	*t*_(54)_ = 0.86, *p* = .396
Targeted professional field (%)	Engineering 44.6 % Educational 12.5 % Marketing 12.5 % Psychology 16.1 % Others 14.3 %	χ^2^_(4)_ = 3.00, *p* = .558
Residential area (%)	64.3 % urban	χ^2^_(1)_ = 0.44, *p* = .506

### 3.2 Program feasibility, acceptability, and satisfaction

The dropout rate was 36 %. The first module was completed by 96.4 % of the enrolled participants and dropped to 64.3 % by the end of the program. The quality of the assignments' completion was high among the protocol-compliant participants, with means above 4 out of 5 for each module. For the dropouts, there was insufficient data for such analyses since more than half of them completed only the first module. iJobs's system usability was good to excellent, with a mean of 84.86 points out of 100 (Bangor et al., 2009). Participants reported high overall satisfaction with the program and information quality, and most of them would recommend iJobs to a friend. The descriptive statistics for all the quantitative satisfaction items are presented in Table 3.

**Table 3:** Descriptive statistics on feasibility, acceptability, and satisfaction with the program.

	Modules				
	1	2	3	4	5
% of completers *per* module					
All enrolled participants (*N* = 56)	96.4 %	76.8 %	76.8 %	69.6 %	64.3 %
Protocol-compliant participants (*N* = 36)	94.4 %	94.4 %	100 %	100 %	94.4 %
Quality of assignments' completion (min. 1 – max. 5) – *M*(*SD*) (*N* = 36)	4.17 (1.24)	4.03 (1.27)	4.09 (0.85)	4.09 (0.91)	4.03 (1.17)


	Mean (*SD*) *N* = 36	Minimum (%)	Maximum (%)
System usability	84.86 (10.50)	60 (2.8 %)	100 (5.6 %)

Satisfaction with the program:			
Overall satisfaction with iJobs	4.33 (1.14)	1 (2.8 %)^b^	5 (61.1 %)^c^
Informations' quality	4.42 (0.99)	1 (2.8 %)^b^	5 (61.1 %)^c^
Satisfaction with iJobs's tempo^a^	2.89 (0.62)	1 (2.8 %)^b^	4 (69.4 %)
Number of completed modules	4.89 (0.31)	4 (11.1 %)	5 (88.9 %)^c^
Number of understood modules	4.72 (0.51)	3 (2.8 %)	5 (75 %)^c^
The activities were demanding	2.17 (0.60)	1 (11.1 %)^b^	3 (27.8 %)
iJobs was useful in approaching my problems	3.53 (0.56)	2 (2.8 %)	4 (55.6 %)^c^
iJobs's methods were logical	8.97 (1.40)	3 (2.8 %)	10 (44.4 %)^c^
I would recommend iJobs to a friend	9.42 (1.31)	3 (2.8 %)	10 (69.4 %)^c^
iJobs will be useful in the future	6.03 (1.05)	3 (2.8 %)	7 (44.4 %)^c^
iJobs deserves to be completed	6.39 (0.99)	3 (2.8 %)	7 (63.9 %)^c^
I want to learn more about iJobs's contents	6.06 (1.01)	3 (2.8 %)	7 (44.4 %)^c^
iJobs's objectives match my needs	6.11 (0.97)	4 (5.6 %)	7 (47.2 %)^c^
I liked iJobs's activities	5.94 (1.06)	3 (2.8 %)	7 (38.9 %)^c^
iJobs's activities helped in the learning process	6.08 (1.02)	4 (8.3 %)	7 (47.2 %)^c^

a 3 = appropriate tempo.

b Minimum value.

c Maximum value.

A brief content analysis of the open-ended questions revealed that when they made overall evaluations, most frequently, participants referred to the program as being *very useful* and *reasonably useful*; the most appreciated features were, in descending order of frequency, the *information*, the *applications*, and the *personalized feedback*. When asked about the disappointing aspects, the great majority reported *none*, while five participants made specific mentions (e.g., *too fast pace*, or it was *too slow*, or *aspects not treated in great detail*). In terms of suggestions for improvement, in descending frequency, the majority of participants had *none*, some needed a more in-depth approach to *resume building*, and the *selection interview*.

Therefore, we can conclude that iJobs is a feasible program and was accepted by the participants.

### 3.3 Outcomes (per-protocol and ITT analysis)

As shown in Table 4, increases in inoculation against setbacks, job search self-efficacy, and self-esteem scores from pre to post-intervention were statistically significant. These differences were small to medium in effect size. The increase in self-esteem can also be depicted based on the data of Schmitt and Allik (2005). Specifically, while our sample's baseline (*M* = 29.52) was very similar to the one estimated on the Romanian general population (*M* = 29.54), the post-intervention level (*M* = 30.94) increased at the average level across 53 countries (*M* = 30.85). The decrease in the total score of future career anxiety was also statistically significant. Therefore, hypotheses 1, 2, 3, and 4c were supported. These results were also replicated in the intent-to-treat analysis. No significant differences were found for depression (H4a) and anxiety (H4b). The overall increase in job search behavior between pre-intervention and follow-up was not statistically significant, nor separately for job search intensity and effort.^2^ The within-group effects for all the outcomes are shown in Table 4.

**Table 4:** Descriptive statistics and baseline to post-intervention comparisons for all outcomes (Per-protocol and intent-to-treat data).

Variable	Pre-test *M*(*SD*)	Post-test *M*(*SD*)	*t*-Test	Cohen's *d* (95%CI)
Per-protocol analysis (N = 36)				
Inoculation against setbacks	6.66 (1.65)	7.63 (1.37)	−2.82⁎	0.64 (0.15, 1.12)
Job search self-efficacy	68.05 (16.43)	75.41 (11.41)	−3.05⁎	0.50 (0.16, 0.85)
Job search behaviors overall^a^	43.03 (13.16)	44.18 (12.41)	−0.51	0.09 (−0.24, 0.42)
Job search intensity^a^	32.32 (10.92)	33.62 (10.08)	−0.73	0.13 (−0.35, 0.60)
Job search effort^a^	10.71 (2.90)	10.56 (3.59)	0.20	−0.03 (−0.50, 0.45)
Self-esteem	29.52 (5.17)	30.94 (4.81)	−3.80⁎⁎	0.28 (0.13, 0.42)
Future career anxiety	16.63 (4.42)	15.05 (4.90)	2.47⁎	0.34 (0.06, 0.61)
Anxiety	5.25 (4.03)	5.22 (4.32)	0.05	0.01 (−0.23, 0.25)
Depression	5.77 (4.27)	5.63 (4.62)	0.20	0.03 (−0.26, 0.32)

Intent-to-treat analysis (N = 56)				
Inoculation against setbacks	6.43 (1.46)	7.80 (1.31)	−5.52⁎⁎	0.99 (0.56, 1.42)
Job search self-efficacy	67.23 (14.39)	76.68 (11.62)	−5.15⁎⁎	0.72 (0.41, 1.02)
Job search behaviors overall^a^	40.80 (13.08)	41.75 (13.02)	−0.53	0.07 (−0.20, 0.34)
Job search intensity^a^	30.77 (10.55)	31.55 (10.18)	−0.73	0.13 (−0.25, 0.50)
Job search effort^a^	10.04 (3.14)	9.95 (3.52)	0.20	−0.03 (−0.40, 0.34)
Self-esteem	29.80 (4.86)	30.96 (4.67)	−3.78⁎⁎	0.24 (0.12, 0.37)
Future career anxiety	16.82 (4.22)	14.11 (4.80)	4.57⁎⁎	0.59 (0.32, 0.87)
Anxiety	4.66 (3.80)	5.16 (4.33)	−1.12	−0.12 (−0.33, 0.09)
Depression	5.50 (4.04)	5.96 (4.54)	−0.80	−0.11 (−0.37, 0.16)

a For job search behaviors, the second assessment was at 3-month follow-up (Per-protocol *N* = 34).

⁎ *p* < .05.

⁎⁎ *p* < .01.

Three months after iJobs 55.9 % (*n* = 17) of the available participants for the follow-up screening were employed, 5.9 % were in a selection process for a job (*n* = 4), and 38.2 % were still unemployed (*n* = 13). An unplanned comparison^3^ revealed significantly higher job search effort for participants who found employment (*M* = 11.82, *SD* = 2.72) relative to those who did not (*M* = 8.62, *SD* = 4.13) – *F*_(2, 27)_ = 6.72, *p* = .015, *d* = 0.94. The difference in job search intensity was not significant (*F*_(1, 38)_ = 1.38, *p* = .251, *d* = 0.61); however, the two subsamples are very small (underpowered). Overall satisfaction with the job (*M* = 8.12, *SD* = 1.72; Min = 4, Max = 10) and with the salary (*M* = 7.88, *SD* = 1.65; Min = 5, Max = 10) was high among the employed participants.

## 4 Discussion

Our results showed that iJobs is a feasible intervention and that the participants found it acceptable. The dropout rate was 36 %, close to the mean percentage (i.e., 35 %) reported in the literature on internet interventions (Melville et al., 2010), but higher as compared to the face-to-face delivery of JOBS (e.g., 14 % in the trial of Paver et al., 2020). Moreover, the number and quality of completed modules were high among protocol-compliant participants. The mean score on the System Usability Scale indicates that the participants perceived the online platform as intuitive and easy to use. Participants also reported high satisfaction with the received information and would recommend iJobs to others. Those results are promising from a practical perspective since they suggest that an asynchronous Internet intervention could be appropriate for job seekers. Therefore, iJobs has the potential to become a highly scalable and economical alternative to the traditional JOBS II program.

A secondary objective of the study was to evaluate the extent to which the outcomes of JOBS II are replicated in the online adaptation of the intervention. iJobs could increase participants' self-esteem, inoculation against setbacks, and job-search self-efficacy. Therefore, iJobs has the potential to replicate the effect on two central components of the classic job-search interventions: boosting self-efficacy and stress management (Liu et al., 2014). After the intervention, participants felt more confident in their ability to search for and obtain a job and more capable of dealing with obstacles during the process, including the ones related to the pandemic, since their anxiety regarding obtaining a job in the COVID-19 context decreased.

Beyond their perceptions, more than half of the protocol-compliant participants (55.9 %) actually gained employment three months after the intervention. Even though the overall frequency of job search behaviors did not increase, there was a significant difference between those who found a job and those who were still unemployed. This might indicate that higher job search effort, along with the quality of job search strategies may have played a role in participants' (re)employment (Brenninkmeijer and Blonk, 2011). The participants who found a job were satisfied with their work and their salary, so the job quality was also high.

In terms of mental health, there were no significant differences between measurements for anxiety and depression. The floor effect might explain the results since the participants with clinical scores were excluded from the program, and the pre-intervention mean scores were rather low; although those with severe mental health issues were also excluded from the original JOBS intervention studies.

The current study was an open-label, uncontrolled trial. Therefore, even though the results are promising, the intervention needs further testing in randomized controlled trials to make causal inferences regarding its efficacy and effectiveness. Moreover, the sample characteristics represent another significant limitation of our trial. The participants were young, highly educated, and unemployed for a relatively short period prior to the intervention because the study was promoted through universities and social media platforms. The online format might be less intuitive and intelligible for older participants or people with low education. Also, in the online version of the intervention, a central component of JOBS II is drastically restricted, respectively, social support. In the face-to-face format, participants could interact and share their experiences. Additionally, some exercises (e.g., rehearsing interviews) are more effective in a synchronous format, because they require instant feedback. We substituted the social support by giving participants personalized written feedback after each module. Still, this made iJobs work slightly different than other asynchronous interventions, because the participants had to complete the modules in specific timeframes. The participants would probably benefit from a group chat that allows them to interact anonymously, and we are considering solutions for integrating this component in future studies. Most of the abovementioned limitations will be addressed in a large-scale randomized controlled trial.

### 4.1 Conclusions

Our results suggest that iJobs, an online alternative for the JOBS II program is feasible and was accepted by the beneficiaries. After further testing, iJobs has the potential to become a cost-reduced, easily accessible resource for the unemployed. An asynchronous intervention that allows self-administration might attract a greater number of beneficiaries and therefore increase employability for individuals with hindered access to such face-to-face resources.

## Funding

The work of Andrei Rusu was supported by a grant of the Ministry of Research and Innovation, CNCS - 10.13039/501100006595UEFISCDI, project number PN-III-P1-1.1-PD-2016-1912, within PNCDI III.

## Declaration of competing interest

The authors declare that they have no known competing financial interests or personal relationships that could have appeared to influence the work reported in this paper.

## Data Availability

The data is available on request from the corresponding author.

## Footnotes

1 Unintentionally, *financial strain* was not measured, which is a deviation from the study protocol.

## Footnotes

2 Item analysis for the Job search behaviors scale revealed a significant difference on item 13: “You used resources within companies you worked for (e.g., colleagues) to find potential jobs.” (*t* = −4.12, *p* < .001) and a marginally significant difference on item 12: “You contacted a potential employer by phone.” (*t* = −1.99, *p* = .054).

3 The between-group comparisons were performed controlling for the baseline levels of each type of job search behaviors via analysis of covariance (ANCOVA).
